# Practices and challenges of supervision and delegation in nursing in Africa: a scoping review

**DOI:** 10.1186/s12912-025-03683-9

**Published:** 2025-08-21

**Authors:** Amidu Alhassan, Victoria Leighton Sencherey, Christiana Bobuafor, Gifty Osei Berchie, Susanna Aba Abraham

**Affiliations:** 1 Department of Adult Health, School of Nursing and Midwifery, College of Health and Allied Sciences, University of Cape Coast, Cape Coast, Ghana; 2 Department of Maternal and Child Health, School of Nursing and Midwifery, College of Health and Allied Sciences, University of Cape Coast, Cape Coast, Ghana; 3 Department of Public Health, School of Nursing and Midwifery, College of Health and Allied Sciences, University of Cape Coast, Cape Coast, Ghana

**Keywords:** Nursing supervision, Nursing delegation, Nursing leadership, Healthcare management and Africa

## Abstract

**Background:**

Nursing supervision and delegation are essential components of healthcare delivery, significantly impacting patients’ care and outcomes. In low-resource settings, such as Africa, where shortage of professional nurses has been reported, effective delegation optimises task allocation and nursing workforce use, while supervision ensures safe care and promotes skill and knowledge transfer to less experienced staff. The review aimed to map existing evidence on the practice and challenges of supervision and delegation in the nursing profession across Africa.

**Methods:**

This review adhered to the six steps outlined in the guidelines by Askey and O’Malley. Search was conducted across five main databases, including PubMed, JSTOR, Scopus, Dimensions AI, and Web of Science, using Medical Subject Headings (MeSH) terms for PubMed and refined for other databases. Additional searches were performed in Google Scholar and university repositories. Reference lists of eligible records were also checked for other relevant articles. The last search was conducted in July 2024. Both peer-reviewed and grey literature were included. Search results were screened against predefined inclusion and exclusion criteria, and extraction was done using a data extraction form. Thematic analysis and synthesis were carried out with evidence presented as narrations and summarized in tables.

**Results:**

A total of 16 studies met the inclusion criteria for this review, highlighting both practices and challenges in nursing supervision and delegation across Africa. Effective supervision practices included comprehensive orientation, continuous training, robust monitoring with feedback, and clinical assessments. Key components of effective supervision include clear communication, teamwork, and supportive environments. However, challenges such as inadequate supervisory skills, time-consuming, enormous administrative tasks, and resource constraints were identified. Delegation practices emphasized feedback, continuous instruction, worker empowerment, strength-based task allocation, and task evaluation and verification. Challenges in delegation were primarily related to staffing shortages, skill gaps, role misalignment, insufficient resources, and the complexity of tasks.

**Conclusion:**

Addressing the challenges and improving supervision and delegation practices in nursing is critical for enhancing healthcare delivery in resource-limited settings like Africa. Implementing evidence-based strategies and developing clear policies will improve the effectiveness of supervision and delegation, ultimately enhancing patient care. Collaborative efforts focusing on staff training, resource allocation, and support systems are essential for overcoming these challenges.

## Introduction

Supervision and delegation are critical components in nursing practice, significantly impacting the efficiency and quality of healthcare delivery, particularly in low-resource settings such as Africa. The increasing complexity of healthcare demands that nursing leaders develop effective strategies for supervision and delegation to ensure optimal patient outcomes [1]. The World Health Organization advocates for clear policies and structured delegation protocols to improve healthcare delivery, especially in resource-limited settings, and to prevent the overburdening of nurses [2]. The International Council of Nurses (ICN) and National Council of State Boards of Nursing (NCSBN) recommend that nurses must be trained in delegation and supervision skills to ensure safety, ethical standards, and quality care [3, 4].

Supervision in nursing refers to the guidance, oversight, and monitoring provided by various nursing personnel; including but not limited to nurse leaders, to ensure that staff members adhere to professional standards and deliver safe, effective care [5]. Delegation, on the other hand, involves the transfer of responsibility for performing specific tasks from a registered nurse to another healthcare worker while maintaining accountability for the outcome [6]. In diverse healthcare contexts, especially in Africa, effective supervision and delegation are crucial. Africa faces significant nursing workforce shortages, high disease burdens, and under-resourced healthcare systems, making efficient task allocation and oversight indispensable [7]. Similarly, appropriate delegation allows for optimal use of available resources and personnel, ensuring that tasks are assigned based on competency and expertise [3].

Historically, supervision and delegation in nursing have evolved as nursing roles expanded. Traditionally, nurses were supervised directly by physicians [8], but as nursing became more autonomous, the need for internal supervision and delegation mechanisms within nursing teams became paramount [9]. In Africa, these practices have been shaped by colonial healthcare systems, where task-shifting and delegation were essential to compensate for workforce shortages [7]. Recent reviews highlight several facilitators to improve supervision and delegation in nursing, including the importance of leadership skills, the role of task-shifting, and the impact of cultural dynamics on delegation practices [10–12]. However, cultural barriers and hierarchical organizational structures have been noted to hinder effective delegation in many African countries [11].

Supervision and delegation skills are critical for all nurses, as they are responsible for ensuring the quality and safety of patient care. Nurses with strong supervision skills can mentor and guide junior and or unregulated staff, while effective delegation allows nurses to focus on complex tasks, improving efficiency. According to a study by Kurt et al. [13] in Turkey, nurses with developed supervision and delegation competencies demonstrated higher job satisfaction, reduced stress levels, and improved patient outcomes [14]. Furthermore, these skills were suggested to help mitigate burnout, a significant challenge in African healthcare settings [15]. Studies suggest that a lack of formal training in these areas often leaves nurses struggling to balance their responsibilities [16, 17].

In contrast, research in the global north indicates the benefits of mentorship programs and structured delegation frameworks, which can be adapted to African settings to improve task management and enhance staff capacity [18]. However, many African countries lack comprehensive policies that guide delegation practices. While task-shifting is encouraged in many settings to mitigate the effects of workforce shortages, it often occurs without clear protocols, leading to role ambiguity and increased risk of errors [19]. Furthermore, effective supervision is also limited by the absence of robust regulatory frameworks, making it difficult to ensure consistency in practice across healthcare settings [20].

In the global south, the effectiveness of supervision and delegation in nursing practice may vary across public and private institutions [21]. In public hospitals, supervision is often hampered by resource constraints and overwhelming patient loads, whereas private institutions may have better structures for overseeing tasks [22]. The American Nurses Association (ANA) highlights that delegation should always prioritize patient safety and respect human dignity [9]. In African healthcare settings, ethical concerns often arise when nurses are overburdened or forced to delegate tasks to inadequately trained personnel. Effective supervision helps mitigate these risks by ensuring that all delegated tasks comply with ethical standards and respect patients’ rights [3].

An existing review on delegation in nursing often focuses on high-resource countries, where well-developed regulatory frameworks guide nursing practice [23]. However, limited reviews have explored delegation and supervision practices in African healthcare systems, particularly in low-resource settings. Despite the importance of supervision and delegation in nursing, there remains a significant gap in the literature mapping evidence on their implementation and impact in African healthcare settings. Most studies focus on high-income countries, with limited research addressing the unique challenges faced in Africa, such as task-shifting, cultural barriers, and limited regulatory frameworks [12, 21]. This scoping review aims to map existing evidence on the practices and challenges of supervision and delegation in nursing across Africa. It seeks to identify research gaps, highlight best practices, and propose strategies to strengthen these essential components of nursing practice.

## Methods

The scoping review utilized the six-stage framework established by Askey and O’Malley [24]. The steps of the framework are: 1) Identifying the research question, 2) Identifying relevant studies, 3) Study selection, 4) Charting the data, 5) Collating the data, and 6) Summarizing and reporting the results. The review was reported using the PRISMA-ScR. The protocol for this study has been registered at the Open Science Framework with registration number DOI: 10.17605/OSF.IO/K4Z7G.

## Identifying the research question

This review was guided by the following research questions: 1) What are the practices of supervision and delegation in nursing in Africa? and 2) what are the challenges faced by nurses in supervisory and delegatory roles?

## Search for relevant studies

The search for relevant studies was mainly conducted in PubMed, JSTOR, Scopus, Dimensions AI, and Web of Science. These key databases were selected for their comprehensive coverage of medical and nursing literature essential for capturing diverse research on supervision and delegation in nursing within Africa. With assistance from a qualified librarian, a detailed search strategy was developed using Medical Subject Headings (MeSH) (see Table 1). The search spanned from the inception of each digital database to August 2024. Additional hand search was conducted in Google Scholar and university repositories to ensure exhaustive coverage, including gray literature (thesis). Additionally, the reference lists of eligible studies were checked to identify additional eligible records. This comprehensive search strategy enhances the credibility, relevance, and rigour of the review by ensuring broad coverage across diverse sources, minimising publication bias, capturing context-specific and grey literature, and strengthening the evidence base for informed conclusions.

**Table 1:** Search strategy for search in PubMed

Concept	Keywords/MeSH Terms
#1. Search to identify Supervision	“Organization and administration [MeSH Term]” OR “Supervision” OR “Preceptorship” OR “Direction” OR “Guidance”
#2. Search to identify Delegation	“Delegation [MeSH Term]” OR “Deputation” OR “Legation”
# 3. Search to identify Nursing	“Nursing [MeSH Terms] OR “Nurse”
#4. Search to identify Challenges	“Barriers” OR “Barriers” OR “Problems” OR “Issues “OR “Difficulties”
#5. Search to identify Africa	“Africa [MeSH Term]” OR “sub-Saharan Africa” [MeSH Terms] OR “Algeria” OR “Egypt” OR “Libya” OR “Mauritania” OR “Morocco” OR “Sudan” OR “Tunisia” OR “Western Sahara” OR “Benin” OR “Burkina Faso” OR “Cape Verde” OR “Côte d’Ivoire” OR “Ivory Coast” OR “Gambia” OR “Ghana” OR “Guinea” OR “Guinea-Bissau” OR “Liberia” OR “Mali” OR “Niger” OR “Nigeria” OR “Senegal” OR “Sierra Leone” OR “Togo” OR “Angola” OR “Cameroon” OR “Central African Republic” OR “Chad” OR “Congo” OR “Democratic Republic of the Congo” OR “Republic of the Congo” OR “Congo (Brazzaville)” OR “Congo (Kinshasa)” OR “Equatorial Guinea” OR “Gabon OR “São Tomé and Príncipe” OR “Burundi” OR “Comoros” OR “Djibouti” OR “Eritrea” OR “Ethiopia” OR “Kenya” OR “Madagascar” OR “Malawi” OR “Mauritius” OR “Mozambique” OR “Rwanda” OR “Seychelles OR “Somalia OR “South Sudan” OR “Tanzania” OR “Uganda” OR “Zambia” OR “Zimbabwe” OR “Botswana” OR “Eswatini” OR “Swaziland” OR “Lesotho” OR “Namibia” OR “South Africa”
Overall search strategy	1. 1 AND #2 AND #4 AND #5 NOT Animal 2. 1 AND #3 AND #4 AND #5 NOT Animal Filter activated for English only.

## Study selection

Articles identified through database searches were imported into Mendeley software for reference management and to remove duplicate records. The screening process adhered to the established inclusion and exclusion criteria (See Table 2). Titles and abstracts were initially screened by postgraduate nursing students in the research team, supervised by independent researchers, GOB and SAA, to promote rigor through guided screening. This was done to select eligible full-text records for further screening. Subsequently, eligible full-text records were independently assessed by three researchers (AA, VLS, CB), who screened the records to determine their eligibility for inclusion. To ensure consistency, SAA reviewed the decision by the screeners to resolve any discrepancies and ensure adherence to the review standards. Where inconsistencies were not resolved, a third reviewer was consulted. This structured methodology ensured a thorough and reliable selection of studies, thereby enhancing the validity of insights into the practices and challenges of nursing supervision and delegation across African healthcare settings.

**Table 2:** Eligibility criteria

Study Characteristics	Inclusion criteria	Exclusion criteria
I. *Study design*	i. Studies using: a. Qualitative, b. Quantitative and c. Mixed-Methods	i. Reviews
ii. *Population*	ii. Registered Nurses and Managers	ii. Student nurses, community health workers
iii. *Study focus*	iii. Clinical Supervision and Delegation	iii. Community-level supervision
iv. *Study setting*	iv. Africa	iv. Europe, Australia, the Americas, and Asia
v. *Publication characteristics*	v. Refereed or peer-reviewed articles, original and grey literature	v. Conference papers and commentaries
vi*. Language*	vi. Papers published in English language	vi. Studies conducted in languages other than English Language

## Charting the data

Data extraction was conducted on a template in Microsoft Excel Spreadsheet version 2021, which provided a firm ground for organizing, managing, and analyzing the extracted data. The data were charted on specifically calibrated forms, designed to capture essential information related to supervision and delegation practices and challenges in nursing. These included pre-developed forms with fields for study characteristics, such as author and year of publication, country, study design, population, key findings, and limitations. To ensure accuracy and completeness, the review team pre-tested the forms using a sample of studies. Data extraction was done independently by the authors (AA, VLS, CB). Discrepancies arising during the extraction process were resolved by a consensus procedure involving authors SAA and GOB. Where inconsistencies still prevailed, a third reviewer was consulted. Attempts were made to contact the original investigators when specific information from the published sources was ambiguous or missing. This methodical strategy guaranteed that the process of data extraction was comprehensive and dependable.

## Collating, summarising, and reporting the results

Data from the charted forms was collated into a master spreadsheet, using Microsoft Excel 2021. The assembled data were reviewed for completeness and accuracy to make certain that all relevant information was captured. An inductive thematic analysis was used to derive themes from the data, allowing patterns to emerge naturally from the findings [25]. Major findings were combined to provide an overview of the status of supervision and delegation practices in nursing, especially within the African context. The results were synthesized and presented in a tabular manner, outlining the main themes and challenges identified from the review. The presentation of results was done through a narrative approach supported by tables and charts to facilitate understanding and illustration.

## Consultation exercise

A qualified librarian was also involved throughout the stages of searching and screening to help develop an appropriate search strategy, enabling him to identify relevant studies concerning nursing supervision and delegation within African contexts. Such consultations allowed a comprehensive review to take place through the inclusion of wide-ranging perspectives and expert reviews. Also, this approach enhanced the validity of the review, thus ensuring that the conclusions and recommendations derived from it are well-informed and relevant within the context of nursing supervision and delegation in African health care.

## Results

### Search results

**Fig. 1:**
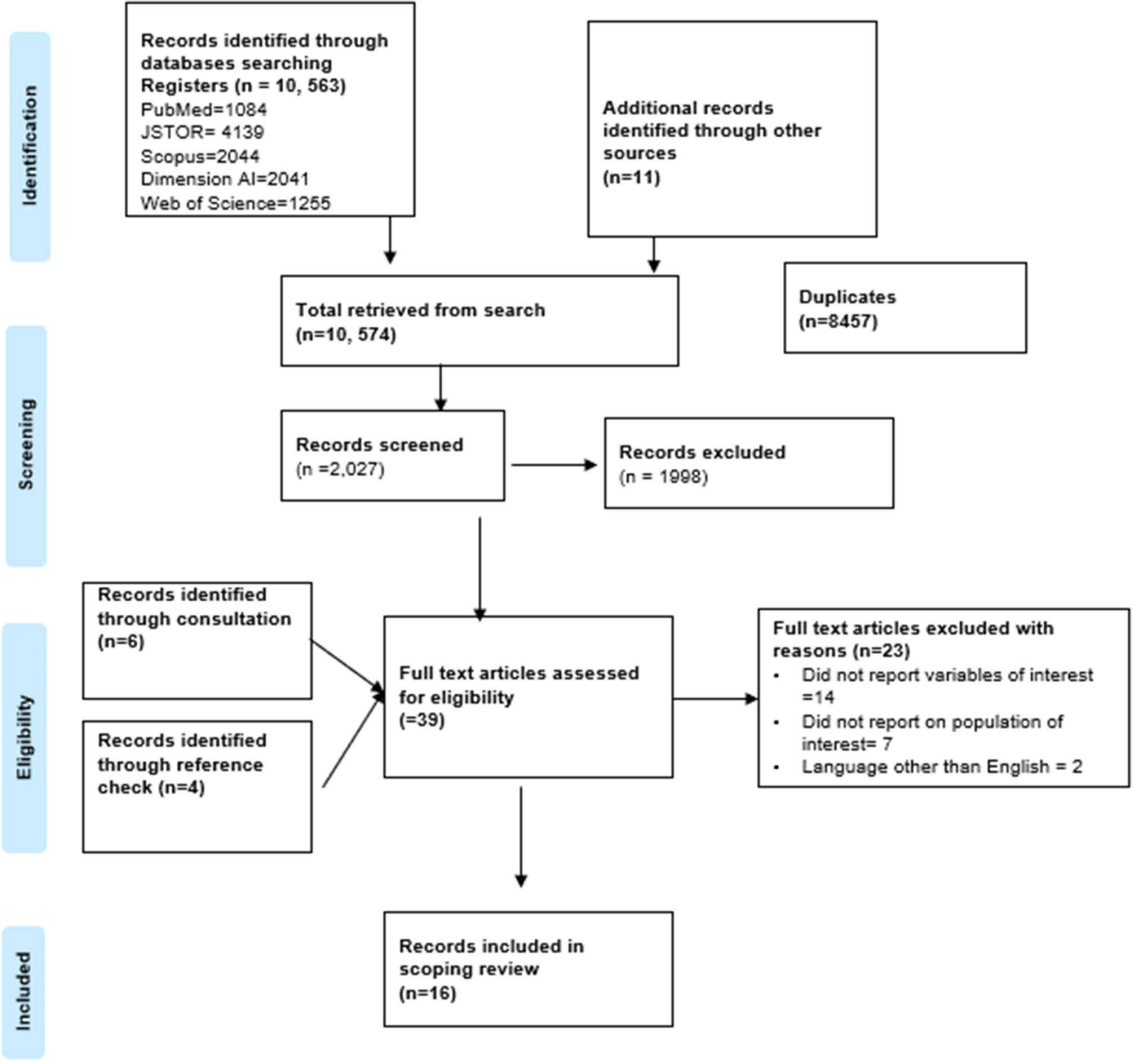
PRISMA flow chart of the search results and screening process

A total of 10,563 records were identified initially, and 11 more were retrieved from other databases. Duplicate records, numbering 8,547, were removed using Mendeley software. After the screening of 2,027 titles and abstracts, 1,998 records were excluded, culminating in 29 eligible records. Thereafter, 39 full-text articles went for further consideration after additional references from bibliographies added 4 more eligible full-text records, while consultations with a digital librarian resulted in 6 more records. From these, 23 records were excluded for specified reasons, leaving 16 records that were included in the review. The PRISMA flowchart is below Fig. 1.

## Characteristics of included studies

**Fig. 2:**
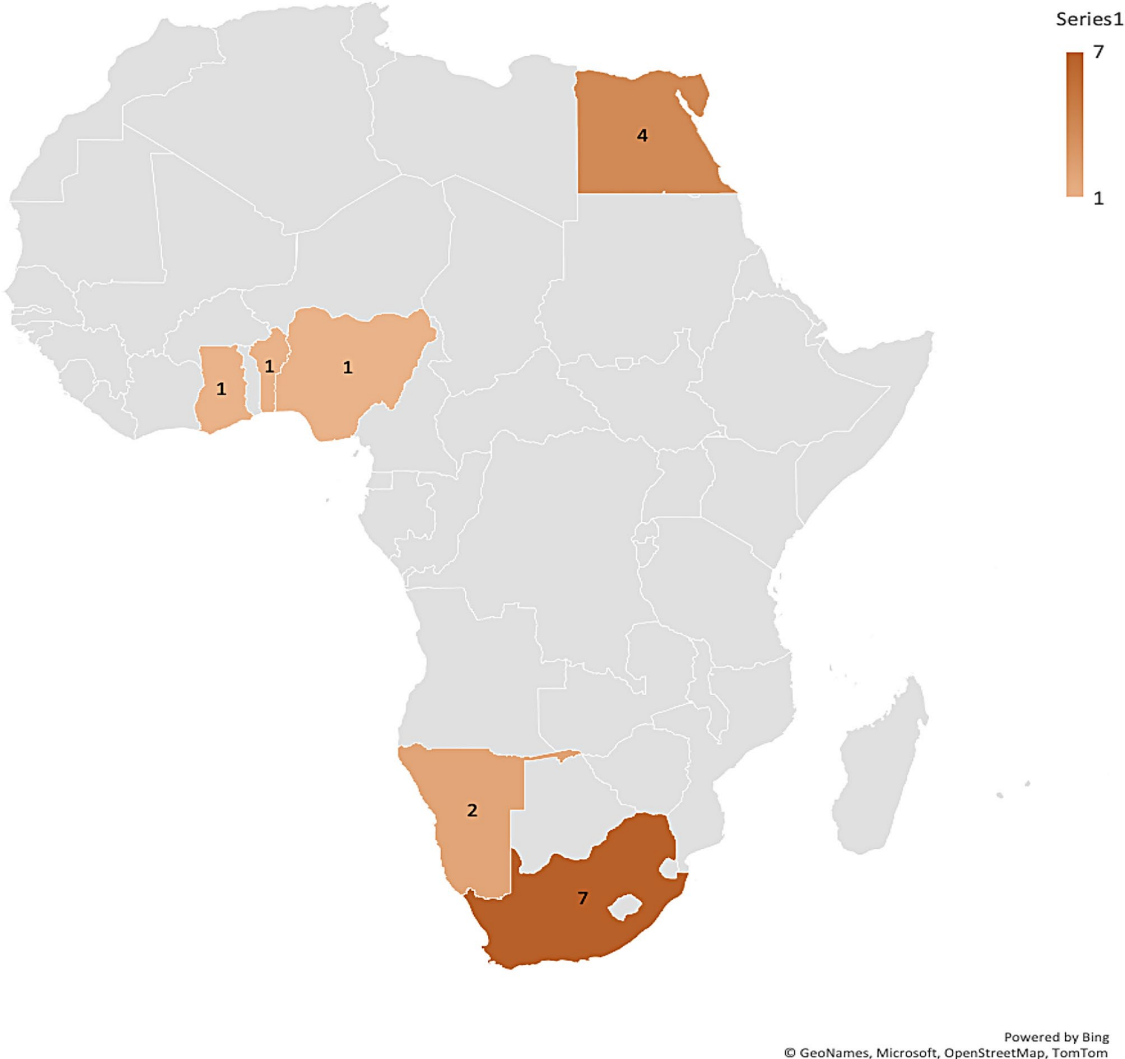
Countries where included studies were conducted

**Fig. 3:**
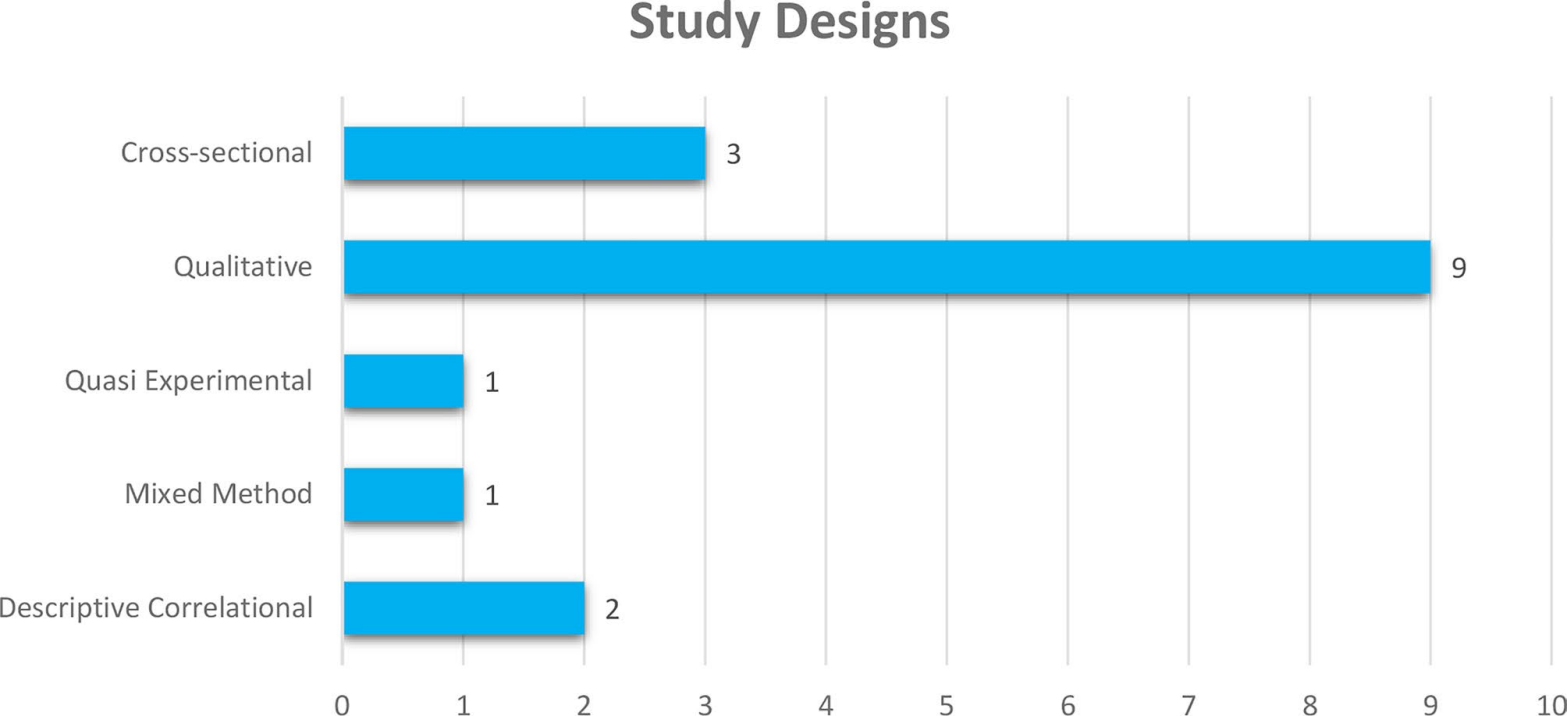
Designs used in the included studies

**Fig. 4:**
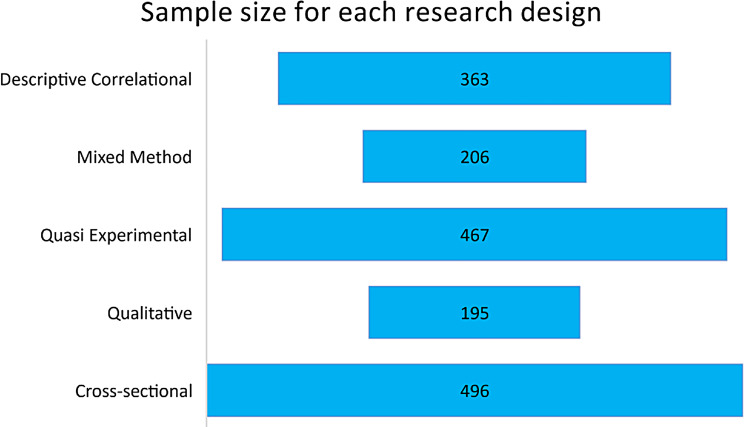
Sample size of included studies according to designs

Most of the included studies were conducted in South Africa [9], followed by Egypt with four studies. Two studies from Namibia were included, while Ghana, Nigeria, and Benin recorded one study respectively. See details in Fig. 2. Out of the 16 included studies, 13 were descriptive, exploratory, qualitative studies. See Fig. 3 for details. A total of 1727 participants were used in the included studies. Details on sample size according to study designs are presented in Fig. 4.

## Practices and challenges of supervision

### Practices of supervision

Studies included in the review reported a diverse range of supervision strategies employed to enhance the effectiveness of supervision practices. Orientation and training emerged as a critical aspect, with initial orientation strategies being widely discussed. Studies highlighted the importance of providing clear explanations, preparing and supporting new supervisors, introducing necessary information, and employing visualization techniques to facilitate effective supervision [26–31]. Also, continuous training was emphasized, including ongoing in-service training, workshops, and opportunities for staff development. Key strategies included training staff members, performing clinical assessments, and capacity building, alongside self-directed learning initiatives [22, 25]. Again, supervision and monitoring practices were thoroughly discussed, with monitoring and feedback being central themes. Effective supervision involves monitoring various activities and reports, providing feedback to the staff member who accepted the delegation, supervising activities, and employing tools and equipment for optimal performance. Debriefing sessions were also identified as a significant practice to ensure comprehensive supervision [6, 22, 27, 28]. Support and guidance emerged as fundamental elements in creating a supportive supervision environment. Studies underscored the importance of good support, a positive atmosphere, trust, and respect, alongside providing guidance and ensuring the availability of clinical supervisors. Documentation and accountability were crucial for maintaining effective supervision. Proper documentation and reporting, including completing compulsory assessments and ensuring accountability, were emphasized as essential practices. Leadership and management strategies were noted as integral to successful supervision. Effective staff management, advocacy, assertiveness, and professional conduct were identified as key leadership practices. Problem-solving and clear objective-setting were also recognized as important for effective decision-making [27, 32], as they enable supervisors to assign tasks with clarity, address emerging challenges promptly, and ensure that delegated responsibilities align with broader organizational goals. Meetings to assess the competence of the staff delegated to were discussed in terms of the supervisors role in providing advice and establishing the competence of community health workers (CHWs) (See Table 3).

**Table 3:** Practices of supervision

Main Themes	Sub-Themes	Authors
Orientation and training	Initial orientation	[22, 23, 33, 34, 35]
Supervision and monitoring	Monitoring and feedback	[5, 22, 25, 27]
Communication and teamwork	Open communication	[27, 28]
	Collaboration and teamwork	[27]
Support and guidance	Supportive environment	[23]
Leadership and management	Staff management and advocacy	[27, 32]
	Problem-solving and decision-making	[35]

### Challenges of supervision

Numerous challenges have been identified in the supervisory role that hamper the ability of supervisors to fulfill their responsibilities effectively. Supervisors face significant challenges in their roles, as highlighted by the issues identified with supervisory responsibilities. Many supervisors reported being unprepared for their responsibilities as clinical supervisors, which impacts their effectiveness [22, 23, 32]. Other challenges included inadequate supervisory skills [36], time-consuming administrative tasks [31], and poor time management due to limited time available for supervisory duties [5, 32]. Similarly, knowledge and training Issues emerged as a significant barrier to effective supervision, with supervisors frequently lacking knowledge about certain equipment [31] and outdated knowledge about supervision practices [37]. There was also a noted lack of guidelines for implementing policies and new practices [38] and inadequate training opportunities [5, 39]. Also, time and resource management challenges were prevalent, with issues related to ineffective time management [37]. Supervisors faced challenges such as a lopsided supervisor-student ratio [37]. Failure to prioritize hospital duties was also noted as a challenge [25]. Likewise, knowledge and supervision skills deficiency included gaps in essential knowledge and clinical skills [27]. Supervisory challenges included inconsistencies in clinical skills instruction, limited supervisor competence [22], and insufficient access to essential information [27], all of which compromise the effectiveness of clinical oversight.

Resource constraints were evident, with shortages in material resources [27] and medical supplies [40]. Additionally, the lack of clinical supervisors [32, 37], and financial constraints [36] critically impair supervision and delegation by limiting oversight capacity, disrupting accountability structures, and restricting the resources needed to support delegated tasks all of which pose risks to care quality and patient safety. Lack of resources and support encompassed various issues including inadequate resources for training supportive supervisors [5] and staff absenteeism [23, 36]. There were also challenges with outdated information [27]. Again, interpersonal and organizational issues included poor communication [25, 37, 39], lack of advocacy [38], and poor interpersonal relationships [37]. Furthermore, environmental and structural issues involved inadequate infrastructure [5, 27], lack of maintenance of teaching equipment [31].

Workload and stress issues included high workloads [34] and stress-related problems such as burnout [30]. Additionally, both supervisors and supervisees reported challenges related to emotional support [38] and anxiety or fear of the unknown [23, 39]. Also, individual and motivational issues encompassed student-related challenges such as favoritism, ignorance, apathy [30], and absenteeism [31]. Issues related to student engagement, preparation for clinical procedures, and professional development were also noted [25–27]. Additionally, administrative and policy issues included failures in policy implementation [38], gaps between theory and practice [23, 37], and resource diversion [29].

In terms of systemic issues, failures in analyzing and resolving problems [38], and ethnic racism [41]. Job conditions such as job insecurity, lack of remuneration were reported [40]. Administrative and procedural challenges included operational issues such as missed deadlines and inadequate assessment procedures [22, 23], as well as logistical challenges like a lack of transport [25, 28]. This scoping review reported educational and supervisory gaps, including a lack of knowledge and skills in supervision [5]. Workplace and interpersonal issues included staffing shortages [27, 32, 37]. Interpersonal conflicts, such as unequal staff treatment and community interference, were also reported [27]. Moreover, individual and motivational issues involved personal challenges like lack of motivation, demotivation, and dishonesty [27, 32, 39], as well as issues of respect and trust [37]. Finally, attitudes and behaviors included negative attitudes towards students and clinical practice [22, 27], unprofessional behavior such as misuse of power and forging signatures [32], and community interference [30]. Challenges related to commitment and inadequate interest were also noted [37].

## Practices and challenges of delegation

### Practices of delegation

Studies on the practices of delegation in nursing reveal several strategies that enhance the effectiveness of this critical process. One prominent theme is instruction, highlighting the importance of providing continuous feedback and structured guidance to unregulated health workers to whom tasks are delegated. This ensures task clarity, promotes accountability, and supports safe clinical outcomes [42]. Another important theme is empowerment, which plays a critical role in delegation practice. When nurses are empowered through a strengths-based approach, they are more confident in assigning tasks, trusting others’ capabilities, and exercising sound judgment in matching responsibilities to team members’ skills. This fosters autonomy while maintaining accountability in clinical care [43, 44]. Furthermore, the theme of assessment, specifically the evaluation of the competence of individuals to whom tasks are delegated, underscores the importance of rigorous evaluation and verification processes to ensure that nursing staff possess the competencies necessary for delivering high-quality care [45].

### Challenges of delegation

The challenges of delegation in nursing are deeply intertwined with supervision, as workforce shortages and resource gaps not only limit who tasks can be delegated to, but also constrain the ability of nurse supervisors to monitor, guide, and support those carrying out delegated responsibilities. Staffing challenges, including a shortage of personnel, significantly impede effective delegation, limiting the ability to assign tasks efficiently [46]. Additionally, skill gaps among nursing staff, particularly the presence of unskilled workers, further complicate delegation, as these individuals may struggle to perform delegated tasks competently, thereby compromising care quality [46]. Furthermore, inadequate resources hinder nurses’ ability to delegate tasks effectively, while the complexity of certain tasks may overwhelm staff, creating additional barriers to successful delegation [47].

## Discussion

### Summary of findings

The findings from this scoping review highlight both the practices and challenges of supervision and delegation in nursing across Africa. Effective supervision often involves structured orientation, clear communication, and regular feedback, supported by various study designs such as descriptive exploratory qualitative designs and cross-sectional descriptive studies. However, challenges persist, including inadequate infrastructure, high staff turnover, and insufficient training, as identified through descriptive exploratory and cooperative inquiry designs. In terms of delegation, effective practices include feedback, continuous instruction, worker empowerment, strength-based task allocation, and task evaluation and verification. Nonetheless, challenges such as staffing shortages, skill gaps, role misalignment, insufficient resources, and the complexity of tasks are significant.

## Practices and challenges of supervision in nursing in Africa

## Practices of supervision

The results revealed essential practices that contribute to effective workforce development, focusing on orientation, continuous training, supervision, communication, teamwork, and leadership. These practices are critical in building a supportive and productive work environment. Initial orientation and continuous training for supervisees, as discussed by Ursula & Hoffman [31], play a fundamental role in preparing nurses for their roles. Providing clear explanations of clinical procedures and introducing additional information through visualization, such as diagrams, demonstration videos, or illustrated checklists, are essential practices that equip employees with the necessary skills to perform their duties effectively. Similarly, continuous training was also revealed to foster capacity building and enhance professional growth, ensuring that staff members stay updated with evolving knowledge and practices [38]. Also, effective supervision and monitoring are key in maintaining accountability and ensuring adherence to procedures. According to Nasiru et al. [29] and Nkomazana et al. [36], regular monitoring, structured feedback, and supervisory visits are key strategies that help ensure consistent and high-quality service delivery in clinical settings. Feedback mechanisms are valuable in guiding performance improvement and fostering a culture of continuous learning. Supervisory visits reinforce accountability and enhance efficiency, especially when supported by reliable transport systems that facilitate timely access to peripheral or hard-to-reach facilities. As noted by Reginah [30] and Kok et al. [34], open communication and teamwork strengthen supervisory processes by promoting transparency, improving feedback flow, and supporting collaborative delegation within clinical teams. Open communication enables information sharing, builds transparency, and promotes staff involvement. Collaboration and teamwork encourage joint responsibilities, role modeling, and cross-learning, which enhance collective problem-solving and empower staff members. This shared learning environment strengthens the organization’s capacity to handle challenges collectively.

A supportive environment, as demonstrated by Donough [44], positively impacts employee morale and performance better supervision. Providing good support, a positive atmosphere, and trust promotes a culture of respect and teamwork. The guidance offered by clinical supervisors, as outlined by Donough & Van der Heever [48], helps build confidence among employees and ensures that they have the necessary resources to succeed. Strong leadership in areas like staff management, advocacy, and problem-solving directly supports supervision and delegation by empowering supervisors to assign tasks effectively, address barriers, and maintain team cohesion. As stated by Reginah [30], effective leadership is a critical enabler of supervision and delegation in African clinical settings, ensuring that staff are well-managed, supported, and motivated to carry out assigned tasks. Within supervisory roles, problem-solving highlighted by Kok et al. [34] is not just a leadership asset but a daily necessity, helping nurse supervisors resolve resource constraints, interpersonal conflicts, and task execution barriers. Assertive leadership supports supervisory nurses in setting clear expectations, making timely decisions, and advocating for their teams, especially in environments where hierarchical constraints and limited resources can hinder effective delegation.

## Challenges of supervision

The thematic analysis reveals several interconnected challenges related to supervisory roles, knowledge, resource management, and interpersonal dynamics in clinical and educational environments. Each theme presents specific issues that collectively highlight systemic gaps and deficiencies that could impact staff. Findings from the analysis indicate a lack of adequate preparation among supervisors for their responsibilities, as highlighted in studies [22, 23]. Supervisors lack adequate skills and time management, which contributes to inefficiency. Similarly, the recurring issue of supervisors being overwhelmed with administrative tasks [28]. Coetzee [32] highlighted the structural imbalance in their roles. This dual burden of clinical duties alongside supervisory responsibilities creates a significant challenge for nurses, limiting their capacity to delegate effectively and maintain consistent oversight, as noted by Hoffman & Daniels [27]. This is further compounded by time constraints, a theme consistent across several studies [5, 33]. Supervisors and clinical staff often struggle with outdated knowledge and insufficient training, an issue that not only affects their supervisory confidence and ability to delegate tasks but also limits their capacity to support others, including students, in the clinical setting. Sah et al. [5] and Nehuku & Amukugo [49] emphasized the outdated knowledge supervisors rely on, while inadequate training on supervision stated a failure in professional development systems [49]. The lack of guidelines for implementing new policies also leaves supervisors and staff unequipped to address modern challenges, causing systemic inefficiencies such as delays in decision-making and fragmented task execution[38]. Resource limitations, both material and human, emerge as a significant barrier to effective clinical supervision. Again, the shortage of medical supplies and human resources, as pinpointed by Magerman [40], Kaphagawani & Useh [50], complicates the ability of supervisors to ensure quality service delivery. Financial constraints and delays in allowance payments, as highlighted by Serapelwane [38], contribute to staff demoralization, which negatively affects nurses’ ability to perform supervisory and delegatory roles effectively. Burnout and dissatisfaction [51] reduce their capacity to provide consistent oversight and mentorship. Furthermore, interpersonal conflicts and poor communication reported by Neshuku [52] and Assegaai & Schneider [33] undermine trust and collaboration, both of which are essential for successful delegation and supervision. Inadequate guidance and a lack of constructive feedback foster a toxic environment for staff and students, making it difficult for nurses to model or enforce professional standards. As Reginah [30] notes, such conditions result in low motivation and negative attitudes toward clinical practice, thereby weakening supervisory influence and the effectiveness of delegated care. Furthermore, poor student engagement and apathy, as noted by Friddah [41], create additional challenges for nurses in supervisory roles, who are expected to motivate, mentor, and provide hands-on guidance. When students disengage, the supervisory process becomes strained, reducing the effectiveness of both supervision and delegation in clinical education. Again, infrastructure inadequacies and the lack of essential equipment, as evidenced by Ursula & Hoffman [31], exacerbate the workload for clinical supervisors, who are already grappling with high demands. Insufficient teaching equipment, Serapelwane [38], and poor facility performance create significant structural obstacles to effective supervision. High workloads, stress, and burnout Serapelwane [38], Coetzee [32] are recurrent themes that reveal a general dissatisfaction among clinical supervisors and staff. These pressures are associated with emotional and psychological distress, as well as anxiety [51]. Donough & Van der Heever [48] acknowledged that the fear of the unknown clinical environment also adds to the mental burden of both supervisors and students, contributing to a decline in their performance.

## Practices and challenges of delegation in nursing in Africa

## Practices of delegation

Our scoping review revealed that organizational practices related to instruction and empowerment consistently emphasized three core themes: feedback and learning, employee empowerment, and assessment mechanisms. These elements collectively contribute to enhanced organizational performance and the professional growth of staff. Feedback loops, as described by Etway & Elewa [43], play a pivotal role in enhancing learning outcomes by fostering a continuous cycle of feedback. This practice enables healthcare workers to receive constructive insights into their performance, promoting adaptive learning and ongoing improvement. By allowing all workers to learn, organizations create an inclusive environment where diverse perspectives and experiences contribute to collective knowledge, aligning with Younes et al [45], who emphasize the importance of accessibility in learning processes. Similarly, continuous instruction, another significant practice identified by Etway & Elewa [43], underscores the necessity of sustained engagement in the learning process. Regularly providing guidance ensures that workers remain equipped to navigate the complexities of their roles, thereby improving both confidence and competence. This is particularly crucial in fast-paced environments where change is constant. Also, Worker empowerment is vital for fostering a positive workplace culture [42, 44]. Empowerment initiatives not only enhance job satisfaction but also motivate employees to take ownership of their responsibilities. This is because empowerment adopts a strength-based approach that focuses on the nurses’ capabilities and not on their deficiencies [42]. This shift in perspective not only boosts morale but also promotes collaboration, as the nurses feel valued for their contributions.

## Challenges of delegation

The analysis of workforce challenges revealed key issues related to staffing, skills, workload, and role clarity, which significantly affect organizational performance and employee well-being. A shortage of personnel, as highlighted by Jennings et al. [46], is a critical problem that limits the ability of organizations to maintain adequate service levels. This shortage creates an over-reliance on a limited workforce, leading to exhaustion and diminished productivity. Moreover, it affects the quality of care or service delivery, as fewer staff members are left to manage an increasing number of tasks. The presence of unskilled or undertrained workers in the clinical workforce poses a significant challenge for nurses in supervisory roles, as it limits their ability to delegate tasks effectively and safely [46]. Skill gaps among team members present a major challenge for nurses in delegatory roles, as they reduce confidence in assigning complex tasks and increase the risk of errors. This often forces supervisors to either perform the tasks themselves or invest time in additional on-the-job training, adding to their workload and stress. This issue is particularly pronounced in sectors requiring specialized knowledge, leading to further strain on existing skilled personnel who must compensate for the gaps. Role misalignment, where employees are assigned duties outside their scope of work, exacerbates workload challenges [53]. This lack of role clarity leads to frustration, burnout, and decreased job satisfaction. Employees may feel overwhelmed by increasing demand, which can lower morale and efficiency. In environments already strained by limited personnel, this issue compounds the negative effects of workforce shortages.

Additionally, resource gaps, as noted by Atta & Fekry [45], highlight the critical importance of adequate financial, technological, or material resources for effective personnel performance. Insufficient resources can severely limit employees’ ability to execute their roles, making it challenging to achieve organizational goals. Hospital administrators, health facility managers, and government health agencies must prioritize investment in critical resources not just to improve operational efficiency, but also to boost staff morale. When healthcare professionals have the tools they need, they can focus on delivering quality care rather than battling constant resource limitations. Furthermore, the complexity of tasks faced by employees can create additional stress and difficulty [47]. Challenging tasks require higher levels of critical thinking, problem-solving, and collaboration, which can overwhelm personnel lacking the necessary training or support. Organizations must ensure that employees are equipped with the skills and support systems needed to navigate these complexities effectively.

## Limitations

While this scoping review employed a structured framework and extensive search strategy, it utilized various study designs, including descriptive exploratory qualitative design, cooperative inquiry group design, cross-sectional descriptive studies, mixed methods, and quasi-experimental designs. Each design provided unique insights but also introduced specific limitations such as small sample sizes and limited generalizability in qualitative studies, self-report bias in cross-sectional surveys, potential researcher influences in cooperative inquiries, and lack of control groups in quasi-experimental designs. The review’s reliance on English-language studies may introduce language bias and exclude relevant research published in other languages. Additionally, publication bias could affect the review’s conclusions, as studies with significant or positive findings are more likely to be published. The quality of the included studies was not assessed, suggesting caution in interpreting the results. Furthermore, the focus on African contexts and timeframes might have restricted the inclusion of broader or more current perspectives, impacting the generalizability of the findings.

## Implications for policy and practice

To address the challenges identified, it is crucial to implement robust training and development programs for clinical supervisors, with a particular focus on effective supervision and delegation. Policies should mandate comprehensive initial training and ongoing professional development, focusing on both theoretical knowledge and practical skills. This will ensure that supervisors are well-prepared for their roles and can handle the complexities of supervision effectively. Institutions should also provide mentorship and regular workshops to update supervisors on best practices and emerging trends, thereby enhancing their competencies and confidence in their roles.

Similarly, efficient time and resource management are essential for effective supervision. Also, policies should formally recognize the demands of supervisory roles by allocating dedicated time and resources for supervision. This includes structuring workloads to allow supervisors to balance clinical oversight with administrative responsibilities. Additionally, integrating time management tools and providing administrative support should be embedded in policy to enhance supervisory effectiveness and reduce burnout. Additionally, institutions must address resource constraints by conducting regular audits and ensuring the equitable distribution of essential resources, such as medical supplies and equipment. This will enable supervisors to perform their duties more effectively and reduce the likelihood of burnout. Also, creating a supportive and collaborative work environment is vital for overcoming supervisory challenges. Policies should promote effective communication, conflict resolution, and a positive workplace culture. Supervisors should receive training in communication skills and interpersonal relations to foster a supportive atmosphere. Additionally, motivational strategies, such as recognition programs and career development opportunities, can enhance supervisors’ engagement and commitment. Addressing personal and motivational issues through tailored support programs will further contribute to a productive and harmonious work environment, ultimately improving the quality of supervision and staff satisfaction.

## Recommendations for future studies

Future studies should prioritize appraising the quality of research on supervisory practices to ensure the rigor and reliability of findings. Additionally, there is a need for longitudinal research that tracks the impact of supervisory training and development programs over time, assessing their long-term effects on supervisory effectiveness, staff performance, and overall clinical outcomes. Furthermore, future research should explore the influence of technological advancements on supervisory practices. Again, cross-cultural and contextual comparisons are essential to understanding how different settings impact supervisory practices. Finally, research should examine how socio-cultural factors, organizational structures, and regional variations influence supervisory effectiveness and resource management.

## Conclusions

The findings of this review reveal both the effectiveness and persistent challenges of supervision in healthcare. While some practices improve clinical outcomes and staff performance, issues such as inadequate training, limited resources, and poor time management continue to hinder effectiveness. Addressing these requires systemic changes, including robust training programs, supportive environments, and improved communication strategies. Key obstacles such as unclear guidelines, knowledge gaps, and resource constraints must be tackled through targeted interventions and policy reforms. Incorporating technology and adapting practices to cultural contexts are also vital. To make meaningful progress, collaboration among researchers, healthcare providers, policymakers, and institutional leaders is essential. This integrated approach can help refine supervision frameworks and ensure they are both context-sensitive and sustainable.

## Data Availability

No datasets were generated or analysed during the current study.

## Appendices

### Appendix 1: Challenges of supervision

**Table:**

Main Theme	Sub-Theme	Authors
Supervisory role issues	Unpreparedness for role	[22, 23]
	Administrative tasks	[31]
	Time management	[5, 32]
	Lack of knowledge on supervision	[5, 22]
	Outdated knowledge	[37]
	Lack of guidelines	[38]
	Lack of training	[5, 39]
Time and resource management challenges	Time management	[37]
	Resource utilization	[31]
		[25, 37]
	Prioritization	[25]
Knowledge and skill deficiency	Knowledge gap	[25, 27, 37]
	Clinical skills	[22]
	Information deficiency	[27]
Resource constraints	Material resources	[28, 54]
	Medical supplies	[40]
	Lack of human resources	[36, 41]
	Financial constraints	[29],)
Lack of resources and support	Inadequate resources	[5, 22, 23, 32, 34, 39, 52, 54]
	Staff support	[27]
	Staff discipline	[38]
Interpersonal and organizational issues	Communication problems	[25, 32, 37]
	Staff relations	[37]
Environmental and structural issues	Infrastructure issues	[5, 27]
	Equipment issues	[22]
Workload and stress issues	Workload issues	[32, 34, 37]
	Stress and burnout	[27]
	Emotional and psychological factors	[23]
	Anxiety, stress and criticism	[23, 39]
Individual and motivational issues	Student-related challenges	[30]
	Student attendance	[31]
	Student engagement	[41]
	Preparation for clinical procedures	[40]
	Clinical exposure	[30]
	Professional development	[25]
Administrative and policy issues	Policy implementation	[38]
	Policy and practice gap	[23, 32, 37]
	Resource diversion	[29]
Systemic issues	Racism	[41]
	Job conditions	[54]
Administrative and procedural challenges	Operational issues	[22, 23]
	Work environment	[25, 28]
Educational and supervisory gaps	Knowledge and skills	[5, 54]
	Teaching materials and methods	[31]
	Supervision quality	[22, 23, 28]
	Supervision and guidance	[25]
	Inadequate evaluation	[22, 27]
	Guidance	[37]
	Observation	[38]
	Colleague influence	[31]
	Training and preparation	[27]
Workplace and interpersonal issues	Staffing and support	[27, 32, 37]
	Interpersonal conflicts	[27]
Individual and motivational issues	Personal challenges	[27, 32, 39]
Attitudes and behaviors	Negative attitudes	[22, 27]
	Unprofessional behavior	[32]
	Community interference	[30]
	Commitment	[37]

### Appendix 2: Challenges of delegation in nursing

**Table:**

Main Themes	Sub-Themes	Authors
Workforce challenges	Staffing issues	[46]
	Skill Gaps	[46]
	Role Misalignment	[53]
Resource Gaps	Insufficient Resources	[47]
Task Complexity	Challenging Tasks	[47]

### Appendix 3: Practices of delegation in nursing

**Table:**

Main Themes	Sub-Themes	Authors
Instruction	Feedback and Learning	[42]
	Continuous Instruction	[42]
Empowerment	Worker Empowerment	[43, 44]
	Strength-Based Approach	[45]
Assessment	Evaluation and Verification	[45]

**Table 1:** Extracted data

Author, Year,Country	Purpose	Study design	Population	Sample size	Supervision practices	Supervision challenges	Delegation practices	Delegation challenges
[31] South Africa	To explore the perceptions of clinical supervisors regarding their preparednessfor the role of clinical teaching	Descriptive exploratory qualitative design	Clinical supervisors	33	Orientation Preparation and supportClearexplanations of procedures Introduces additionalinformation needed regarding the new skillsVisualizationFeedbackMonitoringOpportunitiesTraining workshopsContinuous in-service training	Unprepared for their role as clinical supervisorsAdministrative tasks were time consumingLimited knowledge about certain equipmentFailure to supervise studentsLack of maintenance of teaching equipmentUse of outdated teaching materialsClinical teaching model or framework used by theeducation institution is often not aligned to clinical practice activitiesAbsenteeism of studentsNegative attitudes of studentsWaste of much needed humanresourcesDeadlines and projected assessmentDissatisfaction with the large number of studentsdrained out to preserve rolesIrregularities in teaching clinical skillsNot following the prescribed guidelinesNegative impact of colleagues		
[34] South Africa	To explore and describe experiences of Operational Managers regarding SupportiveSupervision by Local Area Managers in the PHC facilities	Descriptive exploratory qualitative design	Operational Managers	23	AssertivenessProfessional conductTraining of staff members	Lack of joint problem identification and resolutionLack of constructive feedbackPoor facility performance and supervisionFailing to analyze and resolve problemsLack of and unfair staff disciplineLack of guidance on implementation of policies and new guidelinesLack of support by programme coordinators and direct observation of service deliveryStaff shortageDelay in payments of night duty allowanceLack of capturing leave daysStaff absenteeismDelay in rank translationsLack of emotional supportUnequal staff treatmentFrustrations due to lack of appreciationLack of support in cases of litigationsFailure to follow channels of communicationLack of knowledge regarding guidelinesLack of advocacyHigh workloadInsults and blame by community membersLack of essential equipment and inadequate infrastructure		
[5] Ghana	To examine supportive supervision of nurses in the health carefacilities	Descriptive exploratory qualitative design	Management members	3	Monitoring the activities of the nurses on duty Supervising their activitiesTeaching or toeing them on the appropriate activities to do at the wardTools and equipment	Inadequate infrastructure Inadequateresources to train supportive supervisors Inadequate trainingTime issuesLack of knowledge on supervision		
[30] South Africa	To explore the challenges encountered by the professional nurses during supervision of care	Descriptive exploratory qualitative design	Professional nurses	36	TeamworkDebriefingStaff involvementOpen communicationStaff managementRole modeling	Shortage of staffShortage of material resources Inadequate and improper infrastructureNegative attitudeDemotivation and burnout Lack of knowledge, ignorance and apathyPolitics and democratization in nursingCommunity interferenceLack of management and supportFavoritismInadequate exposure to clinical areaLack of student accomplishment		
[43] Namibia	explore and describe the lived experiences of student nursesand registered nurses regarding clinical supervision in themedical and surgical wards in training heath facilities	Descriptive exploratory qualitative design	Registered nursesand nursing students	35		Workload too heavyShortage of staff.Lopsided supervisor-student ratio.Absence of clinical instructors in some wards.Lack of knowledge.Theory and practice gap.Outdated knowledge of supervisors.Ineffective clinical supervision to nursing studentsPoor guidance of nursing studentsPoor interpersonalPoor communicationStock shortages in hospital		
[42] Namibia	To develop, implement and evaluatean educational programme to support registered nurses duringclinical supervision of student nurses in the medical and surgicalwards of the training hospitals	Descriptive exploratory qualitative design	Registered nursesand nursing students	35		Workload Shortage of staffLarge supervisor student ratioNo clinical instructorsLack of knowledge on supervision roleTheory and practice gap Out-dated supervisorsIneffective clinical supervision Poor guidance of students about supervisorsPoor interpersonal relationshipPoor communicationHospital stock shortage		
[29] Nigeria	To investigate the challenges thatstaff nurses in UDUTH faced in the supervision of the clinical activities of the student nurses	Cross-sectionaldescriptive study	Staff nurses	66	CommunicationFeedback	Lack of clinical skillsLack of financialresourcesDiversion of resourceslack of continuing educationLack of skills of nursesFailure to define priorities of the hospital		
[245] South Africa	To determine professional nurses and student nurses' perceptions of clinical supervision	Mixed methods	Professional nurses	467		Shortage of staffIncreased workload Inadequate student nurses' supervisionShortage of material resources correlation of theory into practiceIncreased numbers of student nursesPoor communicationLack of student nurses' involvement Lack of feedbackEthnic racism		
[33] South Africa	To explore the experiences of clinical supervisors, who supervisenursing students from a higher education institution.	Descriptive exploratory qualitative design	Clinical supervisors	8	Advocacy	Time constraintsWorkloadLarge numbers of studentsUnsupportive staffLimited financial resourcesLack of officeLack of medical suppliesUnsuitability of the clinical equipmentUnpreparedness of students for clinical proceduresAbsenteeismLate arrivalsForging supervisor’s signaturesLack of communicationNegative attitude towards studentsUnrealistic work expectationLack of job satisfactionFear of the unknown Unprepared forjob		
[41] South Africa	To explore the clinical supervision experiences of RNs in selected training hospitalswith NS	Descriptive exploratory qualitative design	Registered nurses	10	Clear objectives	No motivation to learnLack of respect for the nursing staffLack of time managementInadequate interest and commitmentLack trust Dishonest behaviorFraudulently signed clinical hoursLack of communicationTheory practice gapInsufficient time at the wardLarge number of studentsUnavailability of supervisorsStaff shortageIncreased workload		
[52] Benin	To explore theinfluence of delegating maternal and newborn counseling responsibilities to clinic-based lay nurse aides on thequality of counseling provided as part of a task shifting initiative to expand their role	Quasi-experimentaldesign	nurse-midwives and lay nurse aide	206				Shortage of personnelUnskilled worker
[27] South Africa	To describe the perceptions of clinical supervisors regarding their preparedness for clinical teaching	Descriptive exploratory qualitative design	Clinicalsupervisors	12	SupportiveSelf-directed learningTraining	Unprepared for roleAnxiety and tensionLack of informationLack of preparednessFrustrationsOutdated informationFailure to supervise studentsTheory practice gapAbsenteeismAssessment deadlinesLarge number of students		
[53] Egypt	To assess barriers of effective delegation as perceived bynursing staff	Cross-sectionaldescriptive study	Nurse supervisors, head nurses, and staff nurses	300				Lack of adequate resourcesDifficult tasks
[49] Egypt	To assess nurses' perception regarding nurse managers' delegation skills andits relation to their job empowerment and loyalty	Descriptive correlation design	Nurses	118			Use feedback loopsGive instructions continuouslyAllow all worker to learn	
[50] Egypt	To investigate the delegation and its relation to job involvement as perceived by staff nurses	Descriptive correlational research design	Staff nurses	245			Empowerment	
[51] Egypt	To determine head nurses' attitudes toward delegationand assess their delegation strategies	Cross-sectionaldescriptive study	Head nurses	130			Allow all worker to learnConcentrates on workers strengthsEvaluationVerificationEncouragement	

## References

[1] Crevacore C, Jacob E, Coventry LL, Duffield C. Integrative review: factors impacting effective delegation practices by registered nurses to assistants in nursing. J Adv Nurs [Internet]. 2023;79(3):885–95. Available from: 10.1111/jan.15430. 36062891 10.1111/jan.15430

[2] World Health Organization. The nursing and midwifery workforce in the African Region: optimizing and accelerating investments for resilient health systems: a regional technical report. 2020;61 p.

[3] International Council of Nurses. Guidelines on Advanced Practice Nursing [Internet]. ICN Regulation series. 2020;44 p. Available from: https://www.icn.ch/system/files/documents/2020-04/ICN_APNReport_EN_WEB.pdf

[4] National Council of State Boards of Nursing. National guidelines for nursing delegation. J Nurs Regul. 2016;7(1):5–14.

[5] Sah C, Antwi-Berko R, Opoku OA, Henry O, Bosiako AA. Exploring supportive supervision of nurses in the health care facilities in volta region, Ghana. J Nurs Res Patient Saf Pr. 2023;3(04):1–10.

[6] American Nurses Association. Principles for Delegation. Publ Progr ANA. 2013;1–16.

[7] Tunny H, Achmad I.The relationship between delegation of medical personnel authority to nurses and nurse job satisfaction. Fundam Manag Nurs J. 2024;7(1):25–32.

[8] American Nurses Association. Delegation in nursing: how to build a stronger team [Internet]. American Nurses Association;2023. Available from: https://www.nursingworld.org/content-hub/resources/nursing-leadership/delegation-in-nursing/

[9] Mbouamba Yankam B, Adeagbo O, Amu H, Dowou RK, Nyamen BGM, Ubechu SC, et al. Task shifting and task sharing in the health sector in sub-Saharan Africa: evidence, success indicators, challenges, and opportunities. Pan Afr Med J. 2023;46:11. 38035152 10.11604/pamj.2023.46.11.40984 PMC10683172

[10] Shore CB, Maben J, Mold F, Winkley K, Cook A, Stenner K. Delegation of medication administration from registered nurses to non-registered support workers in community care settings: a systematic review with critical interpretive synthesis. Int J Nurs Stud. 2022;126:104121. 34896760 10.1016/j.ijnurstu.2021.104121 PMC8803545

[11] Muhammad AN, Danjuma S, Abubakar F, Suleman AM. The Challenges of registered nurses in the clinical supervision of the Bachelor of Nursing Sciences Students At. Sokoto, Nigeria: Usmanu Danfodiyo University Teaching Hospital; 2021.

[12] Crevacore C, Jacob E, Coventry LL, Duffield C. Integrative review: factors impacting effective delegation practices by registered nurses to assistants in nursing. J Adv Nurs. 2023;79(3):885–95. 36062891 10.1111/jan.15430

[13] Kurt Ş, Kose B, Balik N, Ozturk H, Assessment of delegation level in nurse managers, New Trends Issues Proc Adv Pure Appl Sci, 2018 Sep 28, 69–77.

[14] Moradi T, Rezaei M, Alavi NM. Delegating care as a double-edged sword for quality of nursing care: a qualitative study. BMC Health Serv Res [Internet]. 2024;24(1):592. Available from: 10.1186/s12913-024-11054-4. 38715066 10.1186/s12913-024-11054-4 PMC11075185

[15] Achempim-Ansong G, Kwashie A. ansah ofei adelaide maria. Exploring the benefits and challenges of administrative clinical supervision in nursing and midwifery. J Nurs Educ Pract. 2021 Aug 27;12(14).

[16] Atashi V, Movahedi Najafabadi M, Afshari A, Ghafari S. Barriers to effective clinical supervision from the perspective of nurses: a descriptive qualitative study. Nurs Open. 2024 Jan;11(1). 10.1002/nop2.2028 PMC10721941 38268257

[17] Rothwell C, Kehoe A, Farook SF, Illing J. Enablers and barriers to effective clinical supervision in the workplace: a rapid evidence review. BMJ Open. 2021 Sep;11(9). 10.1136/bmjopen-2021-052929 PMC8479981 34588261

[18] World Health Organization. Chronic staff shortfalls stifle Africa’s health systems: WHO study [Internet]. WHO| Regional Office for Africa;2022. Available from: https://www.afro.who.int/news/chronic-staff-shortfalls-stifle-africas-health-systems-who-study#:%7E:text=Africa

[19] Wang T, Tan JYB, Liu XL, Zhao I. Barriers and enablers to implementing clinical practice guidelines in primary care: an overview of systematic reviews. BMJ Open. 2023 Jan;13(1). 10.1136/bmjopen-2022-062158 PMC9827241 36609329

[20] Jones S. Let’s start treating clinical supervision like it’s an essential. Nurs Stand. 2023 Mar 1;38(3):26–27.

[21] Asamoah-Atakorah S, Sanaa Brobbey S, Osei A, Maximous D, Dodoo SW, Benneh Mensah G. The Role of Clinical Supervision in Practical Skill Acquisition of Health Trainees in Ghana. J Heal Med Nurs. 114:2024 Apr 6.

[22] Wilson NJ, Pracilio A, Morphet J, Kersten M, Buckley T, Trollor JN, et al. A scoping review of registered nurses’ delegating care and support to unlicenced care and support workers. J Clin Nurs. 2023;32(17–18). 10.1111/jocn.16724 37149737

[23] Mathebula B, Barnard B. The factors of delegation success: accountability, compliance and work quality. Expert J Bus Manag. 2020;8(1).

[24] Arksey H, O’Malley L.Scoping studies: towards a methodological framework. Int J Soc Res Methodol. 2005;8(1):19–32.

[25] Braun V, Clarke V, Hayfield N, Terry G Thematic Analysis BT - Handbook of research methods in health social sciences. In: Liamputtong P, editor. Singapore: Springer Singapore; 2019. p. 843–60. Available from: 10.1007/978-981-10-5251-4_103

[26] Borragerio F. Clinical Learning Environment and Supervision: student Nurses’ experiences within private health care settings in the Western Cape. By Filomena Borrageiro Health Sciences Professions Research Assignment MPhil in Health Professions Education 2014;(April):1–25.

[27] Hoffman M, Daniels FM. Clinical supervisors’ preparedness for clinical teaching of undergraduate nurses at a University in the Western Cape. Afr J Nurs Midwifery. 2020;22(2).

[28] Mueller C, Vogelsmeier A. Effective delegation: understanding responsibility, authority, and accountability. J Nurs Regul Internet]. 2013;4(3):20–27. Available from.: 10.1016/S2155-8256(15)30126-5.

[29] Nasiru A, Tajuddin M, Maru A. The challenges of registered nurses in the clinical supervision of the bachelor of nursing sciences students at usmanu danfodiyo university teaching. 2021;3(2):96–102.

[30] Reginah RM. Challenges encountered by professional nurses during supervision of care in vhembe district hospitals in limpopo province, South Africa by Raliphaswa Munyadziwa Reginah A dissertation submitted in fulfilment of the requirements of Magister in. Nursing Sci. 2020.

[31] Ursula M, Hoffman M. School of Nursing perceptions of clinical supervisors about their preparedness for clinical teaching at a university in the western cape. 2019;(May). Available from: http://etd.uwc.ac.za/

[32] Coetzee A. The Views of Different Categories of Nurses on Clinical Supervision in the South African Military Health Services (SAMHS). University of the Free State; 2013.

[33] Assegaai T, Schneider H.National guidance and district-level practices in the supervision of community health workers in South Africa: a qualitative study. Hum Resour Health. 2019;17(1):1–10. 10.1186/s12960-019-0360-x PMC6446406 30943986

[34] Kok MC, Vallières F, Tulloch O, Kumar MB, Kea AZ, Karuga R, et al. Does supportive supervision enhance community health worker motivation? A mixed-methods study in four African countries. Health Policy Plan 2018;33:988–98. 30247571 10.1093/heapol/czy082 PMC6263021

[35] Assegaai T, Schneider H.The supervisory relationships of community health workers in primary health care: social network analysis of ward-based outreach teams in Ngaka Modiri Molema District, South Africa. BMJ Glob Heal. 2019;4(6):1–9. 10.1136/bmjgh-2019-001839 PMC6936529 31908861

[36] Nkomazana O, Mash R, Wojczewski S, Kutalek R, Phaladze N. How to create more supportive supervision for primary healthcare: lessons from Ngamiland district of Botswana: co-operative inquiry group. Glob Health Action. 2016;9(1). 10.3402/gha.v9.31263 PMC4921783 27345024

[37] Klerk K Clinical supervision in selected hospitals, Cape Town: reflections on registered nurses lived experiences. 2010;(November).

[38] Serapelwane MG. A supportive supervision framework for operational managers in the primary health care facilities of the North-West Province. 2019.

[39] Neshuku H, Amukugo HJ.Experiences of registered and student nurses regarding the clinical supervision in medical and surgical wards: develop an educational programme to support registered nurses. Int J Med. 2015;3(2):87.

[40] Magerman J Clinical supervisors’ experience of supervising nursing students from a higher education institution in the Western Cape. 2015;(November).

[41] Friddah MR Professional nurses’ and student nurses’ perceptions of clinical supervision in training hospitals of Limpopo Province: south Africa. 2019;(August).

[42] Etway EAE, Elewa AH. Nurses perception regarding nurse managers delegation skills and its relation to their job empowerment and loyalty. J Nurs Heal Sci. Internet]. 2020;9(6):24–33. Available from: www.iosrjournals.org.

[43] Kamphinda S, Chilemba EB.Clinical_supervision_and_support_Perspectives_of_u (1). Curationis. 2019;42(1):1–10.

[44] Donough G. Perceptions and experiences of undergraduate nursing students of clinical supervision. 2014.

[45] Younes HS, Ghallab SA, Mohamed AS head nurses’ attitudes toward delegation and its Strategies. 2017;(April).

[46] Jennings L, Yebadokpo AS, Affo J, Agbogbe M, Tankoano A.Task shifting in maternal and newborn care: a non-inferiority study examining delegation of antenatal counseling to lay nurse aides supported by job aids in Benin. Implement Sci. 2011;6(1):1–14. 21211045 10.1186/1748-5908-6-2 PMC3024964

[47] Atta SM, Fekry NEE. Barriers of effective delegation as perceived by nursing staff in a university hospital. Int J Nov Res Healthc Nurs [Internet]. Available from 2019;6(3): www.noveltyjournals.com.

[48] Donough G, Van der Heever M.Undergraduate nursing students’ experience of clinical supervision. Curationis. 2018;41(1):1–8. 10.4102/curationis.v41i1.1833 PMC6244227 30456982

[49] Nehuku H, Amukugo HJ.Development of an educational programme to support registered nurses during clinical supervision of student nurses in medical and surgical wards in a training health facility, in the oshana region, Namibia. Int J Med. 2015;3(2):80.

[50] Kaphagawani NC, Useh U.Clinical supervision and support: exploring pre-registration nursing students’ clinical practice in Malawi. Ann Global Health. 2018;84(1):100–09. 10.29024/aogh.16 PMC6753308 30873795

[51] Mbonambi MP. Clinical learning environment and supervision of student nurses’ in a private nursing college: a cross sectional study. 2021. 112.

[52] Neshuku H An educational programme to support registered nurses. 2015;(April).

[53] Matsi MM, Lekalakala-Mokgele ES, Madumo MM. Community health workers’ experiences of supervision by nurses at clinics in Limpopo Province. Heal SA Gesondheid. 2023;28:1–10. 10.4102/hsag.v28i0.2330 PMC10778378 38204862

[54] Bellerose M, Alva S, Magalona S, Awoonor-Williams K, Sacks E.Supervision of community health nurses in Ghana: a mixed-methods study on experiences and mentorship needs. Health Policy Plan. 2021;36(5):720–27. 33351910 10.1093/heapol/czaa167

[55] Korang AA, Araf SM, Ali RMN.Delegation and its relation to job involvement as perceived by staff nurses in selected hospital in minia governorate. Minia Sci Nurs J. 2019;006(1):78–85.

